# Medical Imaging Technology for Micro/Nanorobots

**DOI:** 10.3390/nano13212872

**Published:** 2023-10-30

**Authors:** Xuejia Liu, Yizhan Jing, Chengxin Xu, Xiaoxiao Wang, Xiaopeng Xie, Yanhe Zhu, Lizhou Dai, Haocheng Wang, Lin Wang, Shimin Yu

**Affiliations:** 1 State Key Laboratory of Robotics and System, Harbin Institute of Technology, Harbin 150001, China; xuejialiu@hotmail.com (X.L.); jingyizhan218@163.com (Y.J.); x19817739296@163.com (C.X.); greatwxx@163.com (X.W.); xiexiaopeng0421@163.com (X.X.); yhzhu@hit.edu.cn (Y.Z.); dailzh@hit.edu.cn (L.D.); linwang@hit.edu.cn (L.W.); 2 College of Engineering, Ocean University of China, Qingdao 266100, China

**Keywords:** optical imaging, magnetic field imaging, ultrasound imaging, ionizing radiation-based imaging techniques, in vivo environment

## Abstract

Due to their enormous potential to be navigated through complex biological media or narrow capillaries, microrobots have demonstrated their potential in a variety of biomedical applications, such as assisted fertilization, targeted drug delivery, tissue repair, and regeneration. Numerous initial studies have been conducted to demonstrate the biomedical applications in test tubes and in vitro environments. Microrobots can reach human areas that are difficult to reach by existing medical devices through precise navigation. Medical imaging technology is essential for locating and tracking this small treatment machine for evaluation. This article discusses the progress of imaging in tracking the imaging of micro and nano robots in vivo and analyzes the current status of imaging technology for microrobots. The working principle and imaging parameters (temporal resolution, spatial resolution, and penetration depth) of each imaging technology are discussed in depth.

## 1. Introduction

Micro/nanorobots are a new type of robot that can perform self-propelled motion and specific functions at micro/nanoscale. Their structures include spherical, tubular, helical, etc., and they can rely on magnetic fields, chemical reactions, and other means to generate motion and accomplish specific tasks. Since Albert Hibbs and Richard Feynman proposed the idea of using micro/nanorobots for non-invasive disease treatment in 1959 [1], micro/nanorobots have made significant progress in various fields, such as minimally invasive therapy [2,3,4,5], medical imaging [6,7,8,9], and targeted drug delivery [10,11,12]. However, in most scientific research on micro/nanorobots, the observation space for their motion and functionality is mostly limited to experimental setups outside living organisms. There is a lack of research on imaging micro/nanorobots within living organisms, which is essential for clinical applications [13,14]. This paper focuses on the application and development prospects of medical imaging technology in nanorobot observation.

In this paper, we review the state of optical imaging, magnetic field imaging, ultrasound imaging, and ionizing radiation-based imaging techniques critically. Close attention is paid to various imaging modalities, imaging principles, imaging characteristics, and current applications of micro/nanorobots from the perspectives of optical, magnetic, acoustic, radiographic, and integrated imaging, thus pointing out their current limitations and future development directions. Readers are provided with useful instructions and guidelines to conduct future research in the field of in vivo micro/nanorobot imaging. This paper summarizes the characteristics of the above different imaging methods and analyzes the possibility of their application in the direction of micro/nanorobots. At the end of this article, a comparison is made, which provides a certain reference for researchers engaged in related research. In general, the combination of micro/nanorobots and medical imaging technology provides a new means for precision medicine with great development potential.

## 2. Micro/Nanorobot Imaging Techniques and Applications

The combination of micro/nanorobots and medical imaging modalities has a wide range of applications in the medical field. Micro/nanorobots can enter parts of the body that cannot be accessed by catheters or other means, and can be used as carriers to reach the lesion site to release drugs. Medical imaging technology can provide reliable and real-time images for positioning, tracking, and guidance of micro/nanorobots. Medical imaging technology can greatly help people to accurately locate and control micro/nanorobots entering the body. The combination of the two is suitable for a variety of medical fields such as precision medicine. At present, large-scale medical imaging techniques include optical imaging technology, magnetic field-assisted imaging techniques, and ultrasound imaging; in recent years, some composite imaging techniques have been proposed, which combine two or more traditional medical imaging techniques to obtain deeper imaging depth and more biological information. However, this kind of technology is still in the theoretical and research stage and has not yet been applied on a large scale. In order to determine whether various medical imaging technologies are suitable for the localization and navigation of micro/nanorobots, multiple parameters such as imaging depth, imaging time period, and imaging spatial resolution can be used as evaluation indicators. Of course, there is some special equipment for micro/nanorobots imaging, but there are generally problems such as expensive equipment and complex technology, which are far less mature than medical imaging technology.

### 2.1. Optical Imaging Technology

Optical imaging technology captures images by sensing the reflection of tissues under an external light source. It is the simplest and most direct imaging modality among various techniques. Its characteristics include simplicity, low cost, and fast imaging speed [15]. However, due to tissue absorption and scattering of light, its penetration capability is limited.

#### 2.1.1. Fluorescence Imaging Technology

Fluorescence imaging is a common optical imaging technique that consists of a specific wavelength external light source, a carrier with fluorescent dyes, a sensor, and imaging optical devices. Background noise interference can be effectively suppressed by using wavelength filters. Fluorescence imaging utilizes the characteristic of fluorescent dyes emitting light under stimulation by specific wavelength light from an external source, and the matching relationship between the light source and the fluorescent dye is determined based on the Jablonski diagram [16].

**Figure 1:**
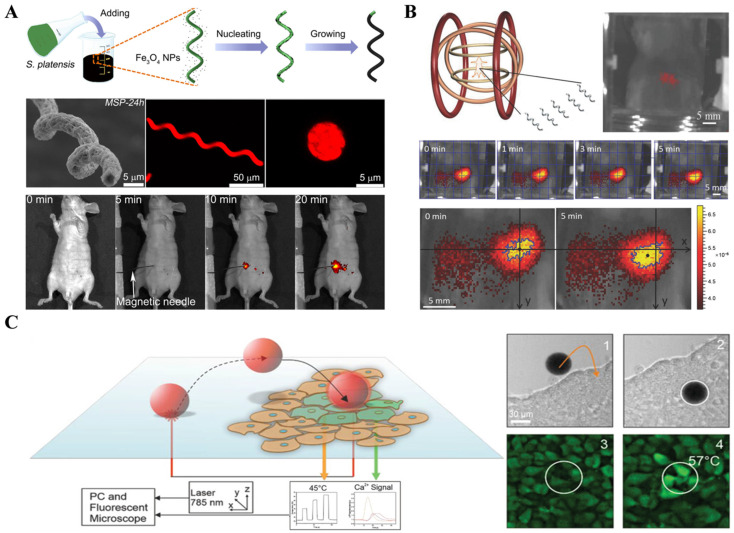
Fluorescence imaging technology. (**A**) Fluorescence imaging technology based on spirulina-based micro/nanorobots. (From [19]. Reprinted with permission from AAAS.) (**B**) Fluorescence imaging of subcutaneous micro/nanorobot tracking in mice. (Reproduced with permission from [21] © 2015 WILEY-VCH Verlag GmbH & Co. KGaA, Weinheim.) (**C**) Schematic representation of a photothermal biostimulation event and corresponding experiment. (Reproduced with permission from [23] © 2023 The Authors. Advanced Healthcare Materials published by Wiley-VCH GmbH).

Fluorescent dyes, such as SU-8 photoresist and quantum dots (QDs), are commonly used as artificial dyes to transfer onto the micro/nanorobot carriers by immersing them in fluorescent solutions. However, most of these dyes have certain toxicity and poor biodegradability and biocompatibility [17,18]. To address this issue, Yan et al. [19] designed a micro/nanorobot based on spirulina, which exhibited fluorescence under specific wavelength light and demonstrated good biocompatibility and biodegradability (Figure 1A). Fluorescence dyes have many advantages, such as low cost, simplicity, high specificity, real-time fast imaging, high planar resolution, sensitivity, and various imaging agent types. Therefore, they are widely used in observing micro/nanorobots. However, their main drawbacks include poor penetration due to light scattering, limited working time due to fluid circulation and metabolism, susceptibility to background light interference, and certain toxicity of most dyes [13,20]. Additionally, fluorescence dyes can produce a broad emission spectrum, leading to overlapping detection ranges and increased difficulty in data analysis, as demonstrated in Servant, A. et al.’s experiment [21]. Yan et al. [19] achieved improved imaging results by immersing micro/nanorobots in fluorescent solutions for 6–72 h before injecting them into subcutaneous tissue and the abdominal cavity of mice. Magnetic probes were used to guide the motion of micro/nanorobots to prevent the diffusion of fluorescent dyes (Figure 1A).

To overcome the limitation of poor penetration in fluorescence imaging, near-infrared fluorescence imaging technology has been widely applied in vivo imaging due to its relatively strong tissue penetration capability. Servant, A. et al. [21] used infrared light and corresponding dyes in the 810–875 nm range to achieve large depth, high precision, and one frame per second imaging in the peritoneal cavity of mice (Figure 1B). Felfoul et al. [22] proposed a strategy to navigate a swarm of fluorescent magnetotactic bacteria (Magnetococcus marinus strain MC-1) to penetrate tumor regions and deliver drugs. Without applying a magnetic field, cells are oriented randomly due to the thermal force-induced Brownian motion or swim toward low-oxygen concentrations. Under a directional magnetic field, the magnetosome (a membranous chain of Fe_3_O_4_ nanoparticles) inside MC-1 provided magnetic torque to drive motion along the magnetic field direction. The results suggested that harnessing swarms of microorganisms exhibiting magneto-aerotactic behavior can significantly improve the therapeutic index of various nanocarriers in the tumor hypoxic regions. Harder et al. verified the biocompatibility and photothermal therapeutic effect of microrobots through fluorescence-enhanced imaging of microrobots (Figure 1C) [23].

#### 2.1.2. Optical Coherence Tomography (OCT) Imaging Technology

Optical coherence tomography (OCT) is an optical interference measurement technique developed in 1991. It consists of a near-infrared broadband light source, a Michelson interferometer, a photodetector, and a spectrometer. OCT can be divided into time-domain OCT and frequency-domain OCT according to scanning imaging modes. Frequency-domain OCT has faster imaging speed and higher sensitivity than time-domain OCT [13].

**Figure 2:**
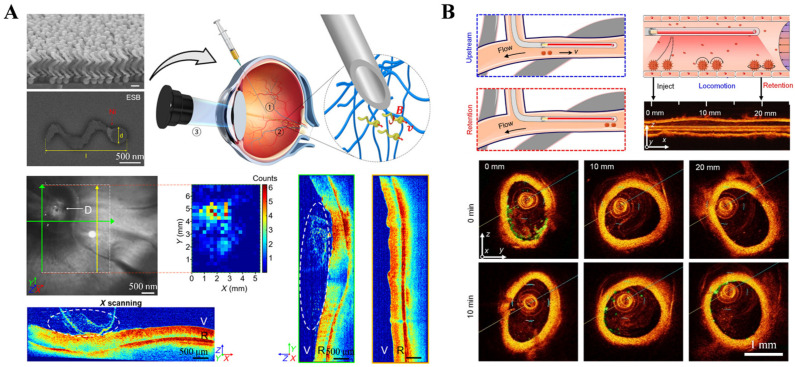
OCT imaging technology. (**A**) OCT monitored the penetration of micropropellers through the vitreous body of the eye. (From [26]. © The Authors, some rights reserved; exclusive licensee AAAS. Distributed under a CC BY-NC 4.0 license http://creativecommons.org/licenses/by-nc/4.0/”. Reprinted with permission from AAAS.) (**B**) Tracking of microrobots’ activity and dynamics based on IVOCT. (From [27]. © The Authors, some rights reserved; exclusive licensee AAAS. Distributed under a CC BY-NC 4.0 license http://creativecommons.org/licenses/by-nc/4.0/”. Reprinted with permission from AAAS.).

As an optical imaging modality, OCT can achieve imaging with micrometer-level penetration depth, featuring non-invasive detection, second-level imaging speed, and micrometer-level resolution [24]. However, its tissue penetration depth is only in the millimeter range, so it is widely used in medical detection and diagnosis in fields such as dermatology, dentistry, and ophthalmology [25]. Wu et al. [26] used clinical OCT technology to monitor the movement of the propellers and confirm their arrival on the retina near the optic disc. The results demonstrated that slippery micropropellers can be actively propelled through the vitreous humor to reach the retina (Figure 2A). Li et al. [27] used the clinical intravascular optical coherence tomography (IVOCT) to track the microrobots’ activity and dynamics in a rabbit jugular vein in vivo, illustrating very effective magnetic propulsion, even against a flow of ~2.1 cm/s, comparable with rabbit blood flow characteristics (Figure 2B). Sun et al. [28] used frequency-domain OCT scanning to track magnetic micro/nanorobots in the mouse portal vein blood vessels, achieving imaging with a tissue depth of 2 mm. Experiments were successfully performed in the portal veins of mice, demonstrating the feasibility of the OCT-enabled navigation strategy. The penetration depth of the OCT imaging system should be further enhanced to help detect the position of microrobots in deep tissues.

### 2.2. Magnetic Field-Assisted Imaging Techniques

#### 2.2.1. Magnetic Resonance Imaging (MRI)

Magnetic Resonance Imaging (MRI) is an imaging technique that utilizes the resonance of hydrogen nuclei in biological tissues with an external magnetic field to produce images. It is also known as magnetic resonance imaging. MRI is well-suited for imaging soft tissues in the human body due to their high hydrogen atom content. MRI has several advantages, including high spatial resolution, strong penetration capability, non-ionizing radiation, and ease of integration with magnetic field-driven robotic systems, making it widely used for observing and navigating micro/nanorobots [13,20].

**Figure 3:**
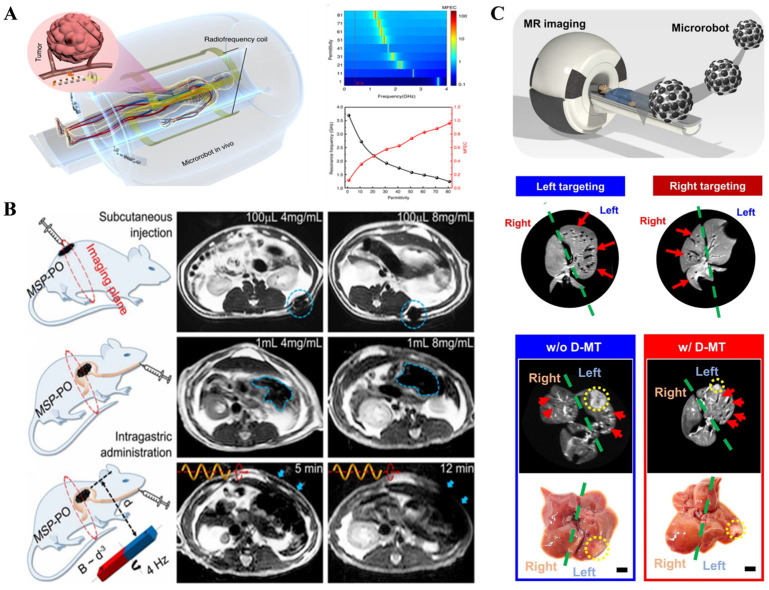
Magnetic resonance imaging (MRI) technology. (**A**) Artificial intelligence microrobot cooperating with electromagnetic imaging equipment for disease diagnosis. (From [31]. Distributed under a CC BY 4.0 license https://creativecommons.org/licenses/by/4.0/. Reprinted with permission from Chinese Academy of Sciences.) (**B**) MRI technology tracks magnetic micro/nanorobots in the mouse gastrointestinal tract. (From [19]. Reprinted with permission from AAAS.) (**C**) Multifunctional microrobot with magnetic resonance imaging for chemoembolization therapy of liver cancer. (From [33]. © The Authors, some rights reserved; exclusive license AAAS. Distributed under a CC BY-NC 4.0 license http://creativecommons.org/licenses/by-nc/4.0/. Reprinted with permission from AAAS.).

When applying MRI to observe micro/nanorobots, contrast agents need to be added to the micro/nanorobots to enhance the imaging effect [29,30]. Superparamagnetic iron oxide nanoparticles (SPIONs) are commonly used as contrast agents due to their simple synthesis, strong magnetic properties, and non-toxicity. Li et al. [31] designed a novel artificial intelligence (AI) microrobot that can respond to changes in the external environment without an onboard energy supply and transmit signals wirelessly in real time. The AI microrobot can collaborate with external electromagnetic imaging equipment to enhance the local radiofrequency (RF) magnetic field, thereby achieving a greater sensing depth and higher spatial resolution. Yan et al. [19] used MRI technology to embed SPIONs on the surface of micro/nanorobot shells and performed MRI scans in specific planes of the mouse digestive tract to track the micro/nanorobots within the gastrointestinal tract (Figure 3B).

MRI imaging imposes certain size requirements on micro/nanorobots and medical contrast agents. According to Shapiro et al.’s research [32], medical contrast agents typically change their magnetic field to about 50 times their own size to form observable images. Therefore, if the size of micro/nanorobots is 500 μm, the contrast agent they carry should be no less than 10 μm to achieve reliable imaging results. Go et al. developed microrobots visualized by real-time X-ray and MRI that can be magnetically guided to tumor blood supply vessels for in vivo transcatheter hepatic chemoembolization. In addition, postoperative degradation of the microrobots can be imaged and tracked using MRI (Figure 3C) [33].

The current major challenge of MRI imaging is the conflict between imaging speed and imaging resolution. According to Pouponneau et al.’s research [34] on magnetic field-driven micro/nanorobots, achieving high-resolution imaging of sparsely distributed micro/nanorobots would increase the imaging time from seconds to minutes, which is not conducive to observing the micro/nanorobots in motion. Currently, there is a lack of optimal strategies to coordinate imaging speed and resolution for micro/nanorobots. Additionally, in prospective MRI imaging–magnetic navigation integrated systems, researchers should consider the magnetic interference of MRI imaging on the magnetic navigation system and use other imaging modalities, such as X-ray or CT imaging, to minimize imaging effects on navigation [35].

#### 2.2.2. Magnetic Particle Imaging (MPI)

**Figure 4:**
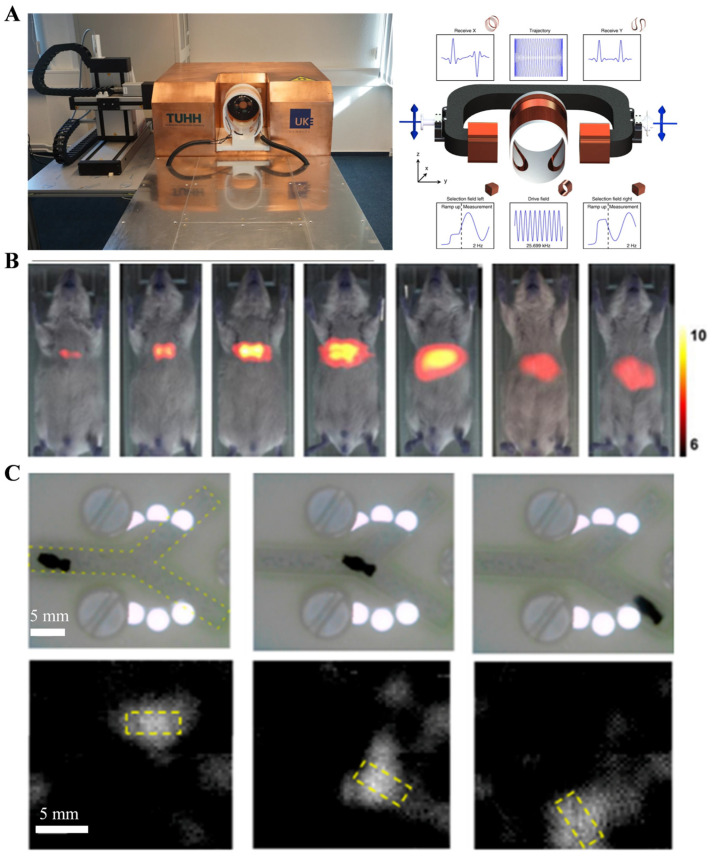
Magnetic field methods for micro/nanorobot observation. (**A**) Appearance and internal magnetic field structure of Magnetic Particle Imaging (MPI) instrument. (Reprinted with permission from [39]. © The Authors. Distributed under a CC BY 4.0 license https://creativecommons.org/licenses/by/4.0/.) (**B**) MPI technology tracks magnetic nanoparticles (NPs) in mice. (From [40]. Distributed under a CC BY 4.0 license https://creativecommons.org/licenses/by/4.0/.) (**C**) MPI technology tracks micro/nanorobots in a vascular model. The dotted yellow box indicates the position of the robot. (Reproduced with permission from [41]. © 2018 Elsevier B.V. All rights reserved.).

Magnetic Particle Imaging (MPI) was first proposed in 2001 by Bernhard Gleich and Jürgen Weizenecker, and it has received widespread attention and rapid development as a new technology in recent years [36]. MPI relies on the Langevin nonlinear magnetic field equation, which describes the response of magnetic nanoparticle (NP) imaging agents to the magnetic field [37]. A strong static magnetic field gradient saturates the magnetization intensity of particles outside the field-free point (FFP), and the high-order harmonic signals that respond to the oscillating magnetic field will be used for imaging [38]. Only particles whose magnetic response is not saturated at the selection field (i.e., at the FFP) will contribute to the detected signal. Complete cross-sectional images of the distribution of magnetic nanoparticles (NPs) are obtained by scanning the sample’s FFP, and the appearance and internal magnetic field structure of the MPI instrument are shown in Figure 4A [39].

MPI relies on magnetic nanoparticle imaging agents and has advantages such as three-dimensional imaging, strong penetration capability, fast imaging speed, high sensitivity, and low cost. It can achieve spatial resolutions on the order of 1 mm and is expected to reach 300 μm with further development of tracers in the future (Figure 4B) [40]. Connell et al. [42] designed a novel magnetic tracer in 2015, achieving high-resolution, high-sensitivity, and long-term tracking imaging in mice. Additionally, MPI technology is suitable for integration with magnetic navigation techniques for micro/nanorobots. Bakenecker et al. [41] designed a spiral microrobot 2 mm long carrying 130 nm magnetic nanoparticles, achieving tracking and navigation of micro/nanorobots in a vascular model using a magnetic navigation system and MPI imaging (Figure 4C).

As an emerging technology, MPI has significant potential for high resolution, high sensitivity, strong penetration capability, linear quantification, real-time imaging, and non-ionizing radiation. However, the current challenges lie in the limited spatial resolution (millimeter level) and the need for further development of specialized tracers, so it has not yet been widely used in clinical applications [40].

### 2.3. Ultrasound Imaging (Ultrasound)

**Figure 5:**
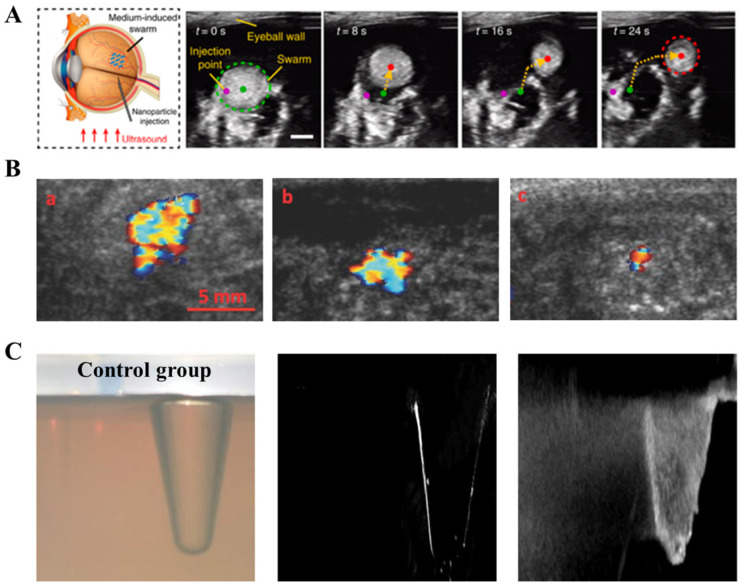
US for micro/nanorobot observation. (**A**) US technology tracks spiral micro/nanorobots in cattle eyes. (Reprinted with permission from [49]. © The Authors. Distributed under a CC BY 4.0 license https://creativecommons.org/licenses/by/4.0/.) (**B**) US of VX2 tumor-bearing rabbits and imaged over the course of 13 days by injecting nanoshells. (Reproduced with permission from [54]. © 2015 WILEY-VCH Verlag GmbH & Co. KGaA, Weinheim) (**C**) Aggregation effect of Janus micromotors under an ultrasonic field. (Reproduced with permission from [55]. © 2021 Elsevier Ltd. All rights reserved.).

Ultrasound imaging is a well-established acoustic imaging technique that uses ultrasound echoes as detection signals to calculate and produce surface images of reflecting objects, reflecting the acoustic properties of human tissues [43]. In B-Mode US, ultrasound contrast agents are typically attached to the surface of micro/nanorobots using specific ligands, and they accumulate specifically in certain locations inside the human body [44,45]. Ultrasound waves are then used to scan the body, and the reflected signals are processed to create images [46]. US has several advantages, including fast imaging speed (up to thousands of frames per second), non-invasiveness, the ability to obtain arbitrary cross-sectional images, deep imaging depth (approximately 3–4 cm), low cost, and mature technology [47]. It is widely used in ophthalmology, obstetrics and gynecology, cardiovascular systems, digestive systems, and urinary systems [48]. Yu et al. [49] utilized B-mode US technology to achieve motion tracking and imaging of spiral micro/nanorobots in the eyes of cattle (Figure 5A).

The current technical challenges of US for observing micro/nanorobots are as follows: First, micro/nanorobots currently have limited capability to detect ultrasound reflections, resulting in small reflected signals, which leads to poor spatial resolution and imaging contrast [50,51,52]. Therefore, US is mainly used to observe micro/nanorobots in tissues with poor echo capability to achieve better contrast [53]. To address this issue, Liberman et al. [54] focus on combining ultrasound contrast agents with the structure of micro/nanorobots to enhance resolution and imaging contrast, achieving twice the brightness of ultrasound contrast imaging (Figure 5B). Feng et al. [55] manufactured a Janus micromotor carrying metallic magnesium, which can produce bubbles as contrast agents in an acidic environment and undergo aggregation under an external 54 kHz ultrasonic field, enhancing US and targeted transportation. This improved the medical imaging observation of micro/nanorobots (Figure 5C). Secondly, US has poor penetration capability in cavities and is negatively correlated with spatial resolution. It has been reported [56] that to achieve a resolution of 100 μm using US at a frequency of 15 MHz, the tissue penetration depth is only about 3–4 mm, making it unsuitable for imaging in deep tissues and organs. Sánchez et al. developed a platform capable of submillimetric positioning accuracy and achieving motion control of self-propelled microjets using B-mode ultrasound images, which addressed the encountered challenges associated with the use of microjets and ultrasound images [57].

### 2.4. Ionizing Radiation-Based Techniques

Ionizing radiation-based imaging relies on high-energy electromagnetic waves with wavelengths ranging from 10 to 100 nm. These techniques have strong penetration capability and high spatial resolution, but the radiation can cause damage to biological tissues. Ionizing radiation-based imaging can be divided into two categories based on the wavelength: X-ray-based imaging and gamma-ray-based imaging. X-ray-based techniques include Computed Tomography (CT) and Fluoroscopy, while gamma-ray-based techniques include Positron Emission Tomography (PET) and Single Photon Emission Computed Tomography (SPECT).

**Figure 6:**
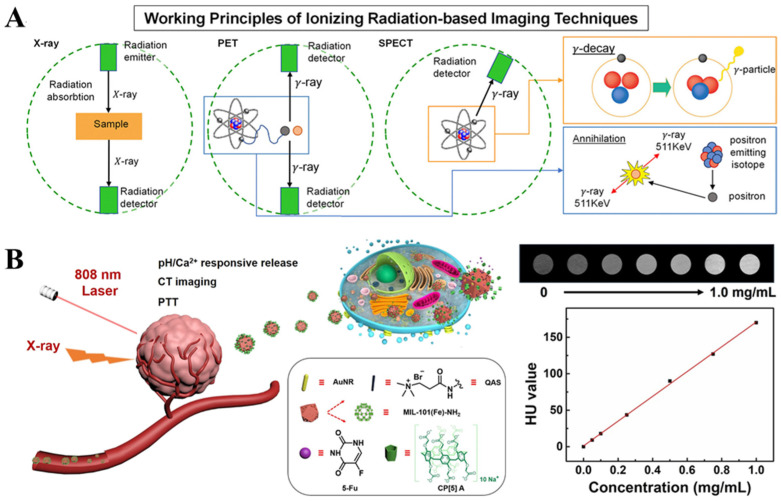
X-ray-CT imaging: (**A**) X-ray, PET, and SPECT working principle schematization. (Reprinted (adapted) with permission from [13]. Copyright 2020. American Chemical Society.) (**B**) Guidance of encapsulating gold nanorods using X-ray CT. (Reproduced with permission from [64]. © 2021 Elsevier Ltd. All rights reserved.).

CT imaging is a common medical imaging technique based on X-rays. The principle of CT is to place the imaging object between an X-ray source and an X-ray detector [58]. Different components and thicknesses of the object will absorb X-rays to varying degrees, causing the detector to receive weaker X-ray signals from corresponding parts. Thicker tissues with stronger absorption capabilities will result in weaker signals received by the detector, and a computer processes the received signals to generate an image (Figure 6A) [13]. By rotating the X-ray source and detector from 0 to 360 degrees, signals will be created and later processed by the computer, where 3D images will be obtained. The human skeletal system, with its thickness and high calcium content, strongly absorbs X-rays, making it especially suitable for CT imaging [59]. Similarly, in the application of imaging micro/nanorobots, allowing micro/nanorobots to carry metal particles as contrast agents enables clear imaging of the micro/nanorobots [60,61]. Jeong et al. [62] used X-ray imaging technology to achieve motion tracking and imaging of micro/nanorobots in the abdominal aorta to the right/left external/internal iliac artery of rabbits.

The current drawback of CT imaging is the need to increase imaging speed. To ensure a frame rate of 25–30 frames per second with a constant total radiation dose, the radiation intensity emitted per frame should be about 0.1% of the total dose, resulting in lower spatial resolution for CT imaging [63]. Therefore, if CT imaging is used for real-time tracking and navigation of micro/nanorobots, it is necessary to control exposure time and increase image acquisition speed while maintaining spatial resolution. Gao et al. developed a stimuli-responsive theragnostic nanoplatform to guide the encapsulating gold nanorods using X-ray CT imaging technology, achieving tumorous microenvironment-triggered drug release and effective chemo-photothermal synergistic therapy (Figure 6B) [64].

**Figure 7:**
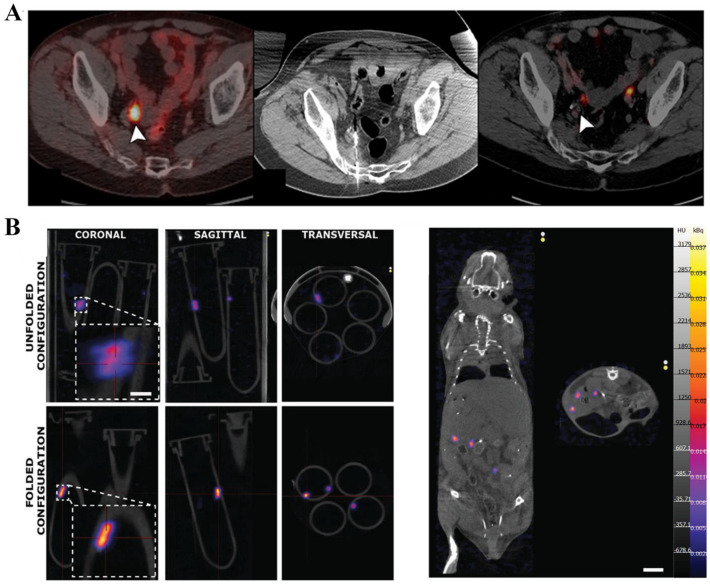
PET-CT and SPECT imaging: (**A**) PET-CT images after prostatectomy with recurrent prostate cancer in a right iliac chain lymph node treated with microwave ablation. The arrow represents the optical imager. (Reproduced with permission from [67] © SNMMI.) (**B**) in vitro SPECT images of microrobots in radiolabeled microrobots subcutaneously injected in mice. (Reproduced with permission from [69]. © 2019 WILEY-VCH Verlag GmbH & Co. KGaA, Weinheim).

PET and SPECT are both gamma-ray-based imaging techniques, also known as nuclear medicine imaging [65,66]. Nuclear medicine imaging involves injecting radioactive elements into the body and placing multiple detectors outside the body to receive the radiation signals from inside the body. The use of PET and MRI also enables molecular-guided interventions with a growing number of novel agents, which can improve the localization of certain tumors. For example, PET/CT with prostate-specific membrane antigens can facilitate the treatment of oligometastatic prostate cancer not otherwise seen with conventional imaging (Figure 7A) [67]. A computer calculates and determines the position of the radioactive target element. PET tracking technology mainly uses radioactive light elements such as 11C, 13N, and 18F, with half-lives ranging from 10–100 min, while SPECT mainly uses radioactive heavy elements such as 99mTc and 123I, with longer half-lives ranging from 6–13 h. Nuclear medicine imaging is strong in anti-interference ability since it only has a radioactive source inside the body, making it particularly suitable for combining with CT for in vivo tracking and imaging. Shi et al. used the PET/CT to monitor the presentative 18F-FDG of mice treated by Bif@PDA-PTX-NPs biohybrids and demonstrated that Bif@PDA-PTX-NPs exhibited a stronger anti-tumor effect and significantly prolonged the survival of tumor-bearing mice [68].

Iacovacci et al. [69] used SPECT technology to inject micro/nanorobots carrying radioactive isotopes into the abdominal cavity of mice and achieved high spatial resolution imaging within the mice (Figure 7B). Vilela et al. [70] marked 12 μm sized tube-shaped micro/nanorobots with 124I and achieved in vivo imaging within 15 min. The limitations of nuclear medicine imaging are slow imaging speed, making it difficult for real-time imaging and tracking navigation, high cost, short half-lives of radioactive isotopes, which restrict long-term tracking, and potential side effects of radioactive elements on biological organisms. It is worth noting that the radiation dose of nuclear medicine imaging in micro/nanorobot tracking is less than that of CT imaging, as the number of radioactive isotopes in micro/nanorobots is minimal, whereas CT imaging radiation originates from an external source with a larger radiation area, and the X-ray source needs to rotate for 3D imaging, resulting in a higher radiation dose than nuclear medicine imaging [13].

### 2.5. Composite Imaging Techniques

Composite imaging techniques combine multiple imaging methods, such as optical, acoustic, and magnetic field imaging, to leverage the advantages of each technique and have wide application prospects. Currently, common composite imaging techniques include photoacoustic imaging (PAI) and magnetomotive ultrasound Imaging (MMUS).

#### 2.5.1. Photoacoustic Imaging (PAI)

Photoacoustic imaging is a rapidly developing medical imaging method based on the photoacoustic effect [71,72,73]. The photoacoustic effect refers to the phenomenon where biological tissues absorb energy and undergo thermal expansion when irradiated with pulsed laser light, resulting in pressure changes and the generation of acoustic waves. By experimentally determining the optical, thermal, and elastic properties of different tissues, the characteristics of tissues in the acquired image can be analyzed [74].

Photoacoustic imaging has the following advantages: it combines the strengths of optical and acoustic imaging, with high spatial resolution and relatively deep penetration; it can analyze the chemical composition of tissues [75,76]; it requires low energy and can generate large signals, saving energy and causing minimal tissue damage; and it achieves high precision for images of various sizes [77]. However, its disadvantages include immature image generation algorithms and relatively limited penetration compared to electromagnetic radiation-based imaging techniques such as magnetic resonance imaging (MRI) [78].

Currently, photoacoustic imaging is used in various forms: photoacoustic tomography (PAT) for area scanning imaging, Photoacoustic Microscopy (PAM) for point-by-point scanning microscopic imaging, and Photoacoustic Endoscopy (PAE) mainly used for gastrointestinal and cardiovascular examinations [56,79].

**Figure 8:**
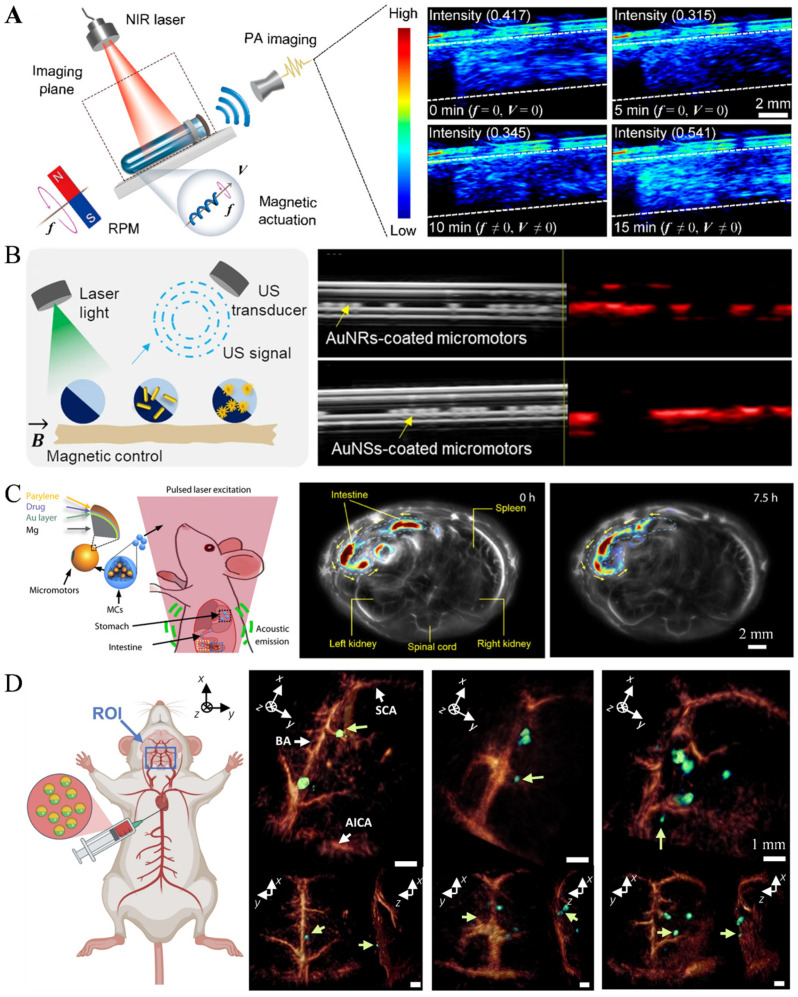
Photoacoustic imaging: (**A**) PA image tracking of microrobot swarm in a plastic tube. (Reprinted (adapted) with permission from [80]. Copyright 2020. American Chemical Society.) (**B**) PA imaging of AuNRs-coated and AuNSs-coated micromotors. (Reprinted with permission from [81]. © The Authors. Distributed under a CC BY 4.0 license https://creativecommons.org/licenses/by/4.0/.) (**C**) PA imaging of micro/nanorobot in a mouse intestine. (Reproduced with permission from [82]. Copyright © 2019, The American Association for the Advancement of Science) (**D**) the obtained optoacoustic tomography images of the Lipo-ICG–coated microrobots (green arrow) in mice. (From [83]. © The Authors, some rights reserved; exclusive licensee AAAS. Distributed under a CC BY-NC 4.0 license http://creativecommons.org/licenses/by-nc/4.0/”. Reprinted with permission from AAAS.).

Based on dopamine-driven PA imaging, Xie et al. achieved non-invasive in vivo motion tracking of a microswimmer swarm and combined ex vivo fluorescence diagnostics and photothermal therapy for multi-drug resistant Klebsiella pneumoniae infections (Figure 8A [80]). Aziz et al. study the Janus micromotors with plasmonic nanomaterials to enhance the photothermal conversion efficiency and PA contrast, greatly improving signal in deep tissue due to the presence of PA agents. The motion behavior of the micromotors through a parametric study using closed-loop control with optical feedback was also achieved (Figure 8B) [81]. In the study of drug delivery by micro/nanorobots in the gastrointestinal system. Wu et al. [82] utilized photoacoustic tomography to embed gold nanoparticles on the surface of micro/nanorobots to enhance the reflected signals, enabling the targeted drug delivery trajectory of the micro/nanorobots in the intestine to be tracked (Figure 8C). Wrede et al. combined a high-performance volumetric optoacoustic tomography system with microrobots enhanced via gold conjugation to facilitate the real-time detection of individual circulating microrobots. The high spatiotemporal resolution of the imaging system achieved 3D real-time tracking of microrobots under ex vivo-, in situ-, and in vivo-like conditions (Figure 8D) [83].

#### 2.5.2. Magnetomotive Assisted Imaging

Superparamagnetic nanoparticles, as important tools in biomedicine, allow manipulation under an external magnetic field for various diagnostic and therapeutic applications relying on excellent biocompatibility, controllable small size, and magnetic properties. The magnetically induced motion of superparamagnetic nanoparticles has become a new source of imaging contrast for enhancing several major imaging modalities such as ultrasound, photoacoustic imaging, optical coherence tomography, and laser speckle tracking, achieving high sensitivity in biological events at smaller scales.

Clinic ultrasound imaging has the advantages of low cost, high temporal resolution, and safety, but its performance for more microscopic molecular imaging is limited. Magneto-motive ultrasound (MMUS) combines conventional ultrasound techniques with an external magnetic field and has attracted more interest recently [84,85,86,87,88]. By detecting the vibration information of the particles using ultrasound waves, spatial distribution information of the particles can be obtained. The key steps of this imaging method mainly include the action of a changing magnetic field on magnetic nanoparticles, the detection of vibration signals, and particle center positioning. Magnetomotive ultrasound imaging has stronger penetration compared to optical imaging and is faster in imaging speed with simpler imaging equipment than MRI, making it suitable for various medical applications.

**Figure 9:**
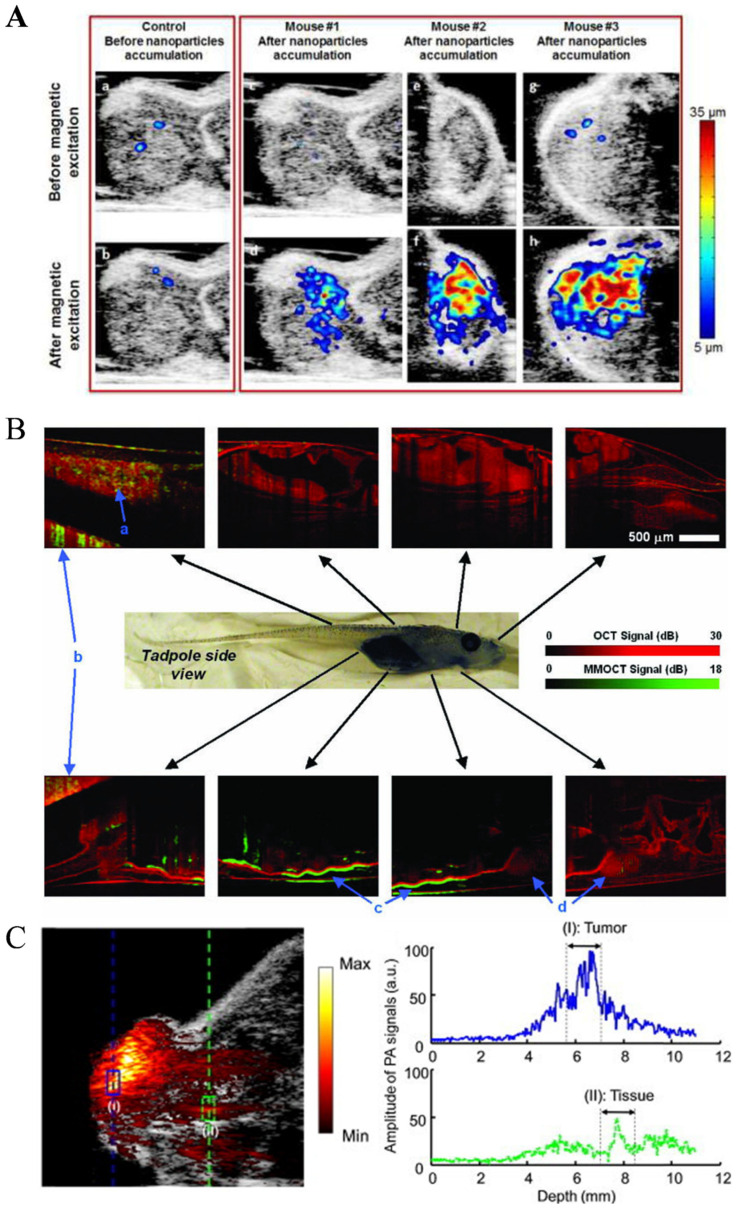
Magnetomotive assisted imaging: (**A**) in vivo US/MMUS images of mice with A431 tumors [89] (Reproduced with permission from [89]) (**B**) in vivo MM-OCT imaging of magnetic nanoparticles in a Xenopus tadpole model fed [92] ([Reprinted/Adapted] with permission from [92] © The Optical Society.) (**C**) magneto-motive PA imaging (MPA) imaging of a tumor in vivo. (Reproduced with permission from [102]. Copyright © 2014 The Authors. Published by Elsevier GmbH).

The core of magnetomotive ultrasound imaging lies in the generation and detection of vibrations. First, magnetic nanoparticles are subjected to magnetic forces, leading to vibrations. By detecting the vibration displacement, vibration information is obtained. The principle involves using ultrasound imaging technology to obtain the echo signals from tissues, allowing the estimation of tissue vibrations. Subsequently, a computer estimates the vibration displacement amplitude in the two-dimensional space and generates a two-dimensional image, which produces an image of the tissue’s internal vibration strength. This allows for precise positioning of magnetic nanoprobes (Figure 9A) [89]. Magnetomotive ultrasound imaging has three imaging modes: B-scan mode, color Doppler mode, and M-mode, which have different imaging effects for the same object.

Similar to ultrasound, the contrast in OCT based on backscattering light is limited in the application of molecular imaging. Magneto-motive OCT (MM-OCT) can detect the motion of magnetic nanoparticles-labeled targets and create high-resolution cross-sectional images when an external magnetic field is introduced [90,91]. MM-OCT enables imaging with higher sensitivity, thus improving the diagnostic value. The molecular-specific MM-OCT has been used in a variety of applications, including tumor imaging in vivo (Figure 9B) [92,93,94], platelet imaging [95,96], and stem cell imaging [97]. In particular, the MM-OCT provided a new method for the assessment of tissue microstructure in vivo avoiding invasive, destructive, histological processing. The mechanical resistance of tissue is also one of the major limitations that MM-OCT needs to overcome, which can limit its use in fluid environments. Another drawback specific to MM-OCT is limited penetration depth.

Photoacoustic imaging relies on the photoacoustic effect to combine the advantages of light and sound; however, the use of exogenous absorbers to distinguish in vivo imaging is particularly important to distinguish background PA signals from endogenous absorbers. In addition, another idea is to use a dynamic contrast mechanism to distinguish absorbers, further suppress background signals, and enhance contrast. MPA imaging is a representative method, including the use of optical and magnetic hybrid nanoparticles. The main feature of generating the MPA image is to use the mask of the PA image to reduce the background noise and obtain the high contrast resolution of PA [98]. In MPA imaging, particle light absorption will generate the PA signal of interest. At the same time, endogenous background light absorbers will also generate photoacoustic signals. Then, the magnetically driven motion of the magnetic particles alters the speckle pattern of the ultrasound image, resulting in an MMUS image [99,100,101]. The MPA image is finally generated by masking the PA image with an MMUS image that suppresses the background PA signal to enhance the detection of photomagnetic nanoparticles (Figure 9C) [102].

## 3. Summary and Future Outlook of Imaging Techniques

The aforementioned optical imaging, magnetic field imaging, ultrasound imaging, and ionizing radiation-based imaging techniques each have their unique characteristics. For example, optical imaging techniques like fluorescence imaging and coherent optical tomography have high temporal resolution and imaging speed but poor penetration capability, making them unsuitable for imaging deep tissues. Magnetic field imaging offers strong penetration capability and high spatial resolution, but it has slow imaging speed, and the compatibility between magnetic driving of robots and imaging must be considered. Ultrasound imaging has fast imaging speed, good penetration capability, low cost, and high safety but relatively lower spatial resolution. Ionizing radiation-based imaging techniques, such as X-ray and nuclear medicine imaging, provide strong penetration and high spatial resolution, making them particularly suitable for tracking and localization. However, they have slower imaging speed, expensive equipment, and potential tissue damage due to radiation. The characteristics of various imaging techniques are summarized in Table 1 [13].

Considering the different imaging scenarios, the selection of appropriate imaging techniques should take into account the following requirements: (1) imaging time; (2) compatibility between micro/nanorobot control methods and imaging techniques (e.g., magnetic driving of robots and magnetic field-based imaging); (3) the dimensions of micro/nanorobot movement, choosing planar or spatial imaging; (4) imaging range; and (5) the effective working time of micro/nanorobots. Sometimes, multiple imaging techniques need to be combined to achieve the required imaging indicators, such as combining fluorescence imaging with ultrasound imaging or combining MRI with fluorescence imaging.

**Figure 10:**
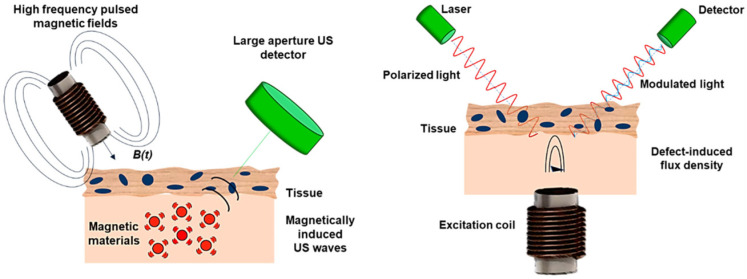
Schematic diagram of acoustic imaging and magneto-optic imaging. (Reprinted (adapted) with permission from [13]. Copyright 2020. American Chemical Society.).

The future development direction of micro/nanorobot imaging technology mainly involves the development of composite imaging techniques. Magneto–acoustic imaging is an emerging imaging technology that utilizes the ultrasound imaging of high-frequency magnetic micro-particles in a magnetic field, combining fast imaging speed and strong penetration capability [103]. Magneto-optic imaging uses the magneto-optic effect of the magnetic field on polarized light for imaging. Composite imaging techniques combine the advantages of multiple imaging techniques and have significant development potential in the future (Figure 10) [13].

In summary, we have discussed and highlighted here the recent medical imaging of microrobots and, at the same time paid great attention to various imaging modalities, imaging principles, imaging characteristics, and current applications of micro/nanorobots from the perspectives of optical, magnetic, acoustic, radiographic, and integrated imaging. Micro/nano robots have great potential value in many medical scenarios such as precision medicine, surgery, in vivo diagnosis, and so on. As an important part of the development of micro/nanorobots, medical imaging technology will also be further developed. Traditional medical imaging technology needs to be further developed, and the research and commercialization of composite imaging technology are crucial for the development of micro/nanorobots. Looking to the future, further research progress and future clinical translation of the in vivo micro/nanorobot imaging require an interdisciplinary collaborative effort among imaging experts, robot scientists, and chemists. With continuing technological innovations, the micro/nanorobots will prove to be of great importance for improving the accuracy and effectiveness of diagnosis and treatment and have broad prospects in the fields of targeted therapy, minimally invasive therapy, precision surgery, and personalized medicine.

## Figures and Tables

**Table 1:** Summary of imaging techniques and future imaging technology outlook. Spatial resolution, temporal resolution (imaging speed), and penetration depth indicators of various images. MPI: Magnetic Particle Imaging; OCT: optical coherence tomography; F: fluorescence imaging; US: ultrasound imaging; PET/SPECT: nuclear medicine imaging; X-ray: X-ray imaging; MRI: magnetic resonance imaging [13] (reproduced with permission from [13]).

Imaging Modalities	Spatial Resolution	Temporal Resolution	Penetration Depth Indicators	Advantages	Disadvantages
Magnetic particle imaging (MPI)	1~10 mm	Millisecond level	Hundreds of millimeters	· High spatial resolution · High temporal resolution · Real-time in vivo 3D imaging	· Requires specific equipment and software · Operation complex · Costly
Optical coherence tomography (OCT)	5~20 µm	Millisecond level	Hundreds of millimeters	· High temporal resolution · High imaging speed	· Poor penetration capability
Fluorescence imaging (F)	100~1000 µm	>1 min	A few millimeters	· High temporal resolution · High imaging speed	· Poor penetration capability
Ultrasound imaging (US)	100~1000 µm	Millisecond level	Hundreds of millimeters to centimeters	· Fast imaging speed · Good penetration capability · Low cost · High safety	· Relatively lower spatial resolution
Nuclear medicine imaging (PET/SPECT)	1~5 mm	>10 s	In the millimeter to centimeter scale	· Strong penetration · High spatial resolution · Suitable for tracking and localization	· Slower imaging speed · Expensive equipment · Potential tissue damage due to radiation
X-ray imaging (X-ray)	10~500 µm	>1 min	>1 cm	· Strong penetration · High spatial resolution · Suitable for tracking and localization	· Slower imaging speed · Expensive equipment · Potential tissue damage due to radiation
Magnetic resonance imaging (MRI)	10~200 µm	>1 min	>10 cm	· Strong penetration capability · High spatial resolution	· Slow imaging speed
